# Coexistence of chronic lymphocytic leukemia and multiple myeloma, do the roots of these entities originate from the same place?

**DOI:** 10.1002/ccr3.951

**Published:** 2017-04-12

**Authors:** Alparslan Merdin, Jale Yıldız, Mehmet Sinan Dal, Merih Kızıl Çakar, Ali Hakan Kaya, Emre Tekgündüz, Fevzi Altuntaş

**Affiliations:** ^1^ Hematology Clinic and Bone Marrow Transplantation UnitDr. Abdurrahman Yurtaslan Ankara Oncology Training and Research HospitalAnkaraTurkey

**Keywords:** Chronic lymphocytic leukemia, coexistent, multiple myeloma

## Abstract

Multiple myeloma is a plasma cell disease, whereas CLL (Chronic Lymphocytic Leukemia) affects mature B‐cell lymphocytes. Even though the coexistence of those two conditions is extremely rare, as both cell types differentiate from the same multipotent stem cells, the clinician should evaluate patients carefully not to misdiagnose such a concomitancy.

A 56‐year‐old male patient was admitted to hospital with severe anemia and lymphocytosis. Physical examination revealed splenomegaly and lymphadenopathy. Laboratory findings revealed lymphocyte: 45,670 mm³(1500–3500), beta‐2‐Mikroglobulin: 12.2 mg/L (0.7–1.8), calcium: 11.6 mg/dL (8.4–10.2), creatinine: 0.89 mg/dL, serum, and urine kappa monoclonal gammopathy positivity. Flow cytometry analysis from peripheral blood (PB) detected CLL (Chronic lymphocytic leukemia); but from bone marrow (BM) aspiration detected MM (multiple myeloma). PB smear showed mature lymphocytes (Fig. 1). Besides, BM smear showed plasma cells (Fig. 2). Bone marrow biopsy showed 80% plasma cell infiltration. Coexistence of CLL and MM is an extremely rare clinical entity. Multiple myeloma is a disease of plasma cells. And CLL is a disease of mature B‐cell lymphocytes. Clonal association in the coexistence of CLL and MM is controversial 1, 2. But plasma cells differentiate from the lymphocytes. And further reports would shed light on this rare concomitant entity.

**Figure 1:**
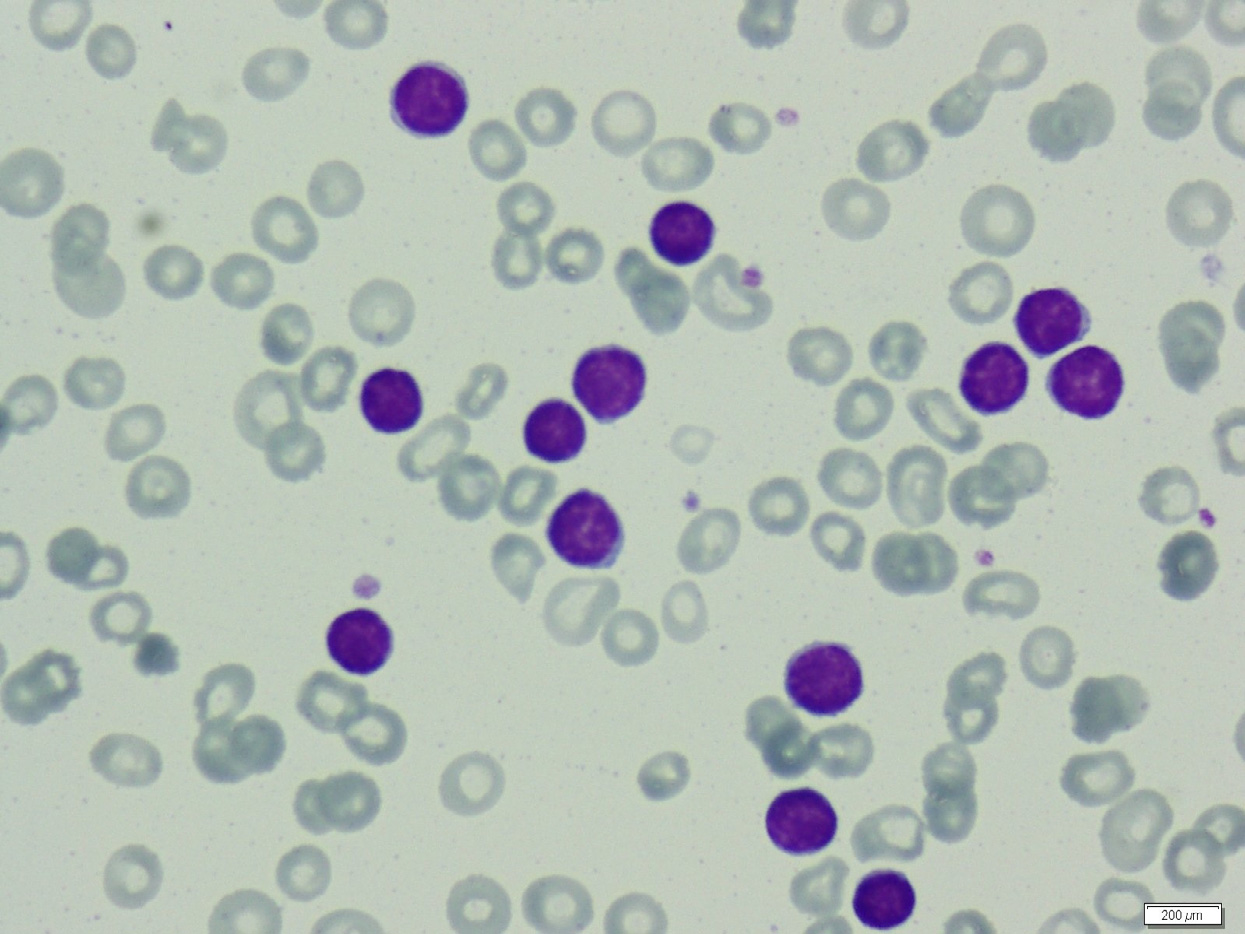
Peripheral blood smear showing mature lymphocytes.

**Figure 2:**
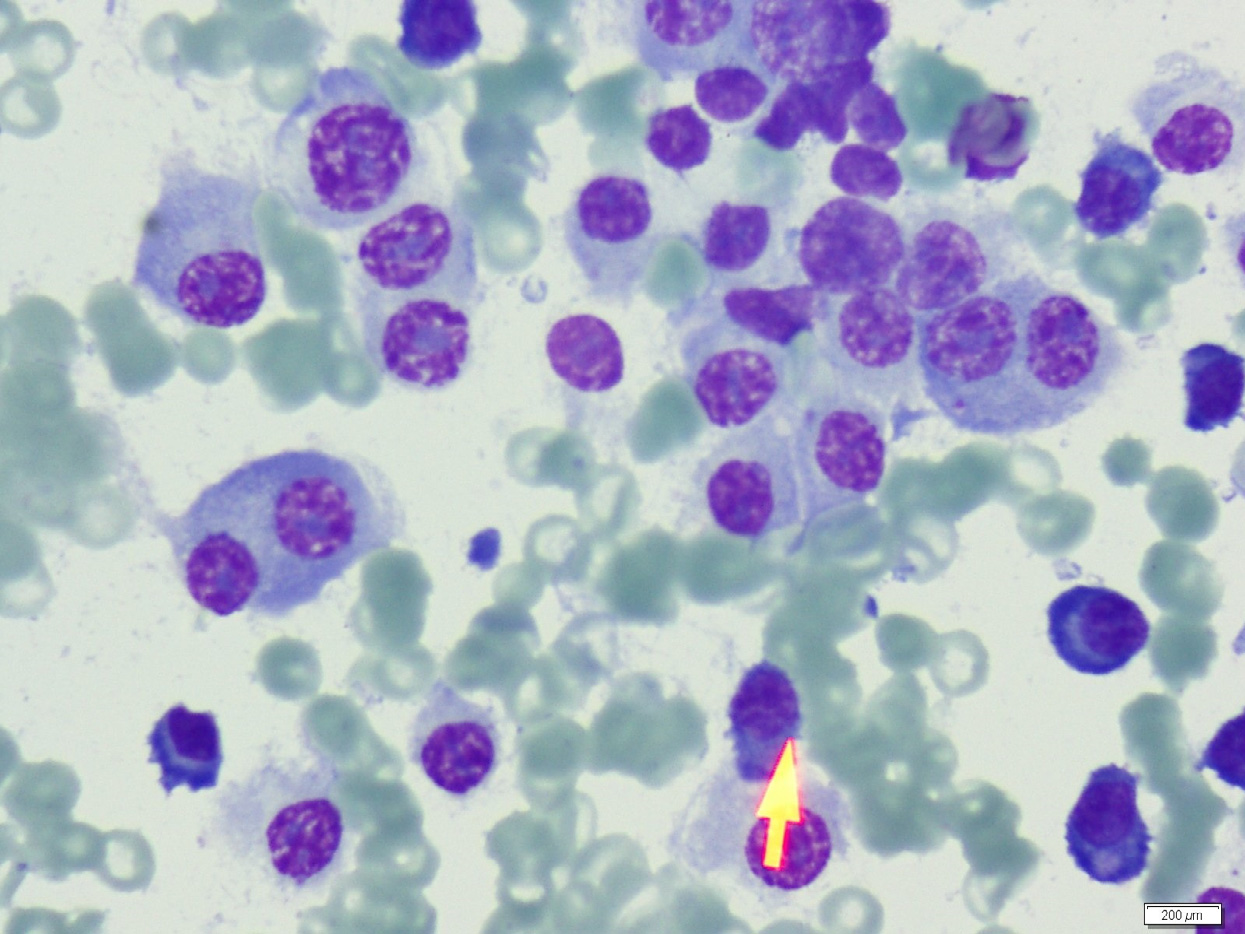
Bone marrow aspiration smear showing diffuse plasma cell infiltration.

## Authorship

AM, JY, MSD, ET, FA: patient follow‐up; AM, JY, MSD, MKÇ, AHK, ET, FA: drafting the article.

## Conflict of Interest

None declared.
